# Runners with better cardiorespiratory fitness had higher prefrontal cortex activity during both single and exercise-executive function dual tasks: an fNIRS study

**DOI:** 10.3389/fphys.2023.1246741

**Published:** 2023-08-14

**Authors:** Bowen Liu, Jingxuan Yu, Jinlong Wu, Yifan Qin, Wen Xiao, Zhanbing Ren

**Affiliations:** ^1^ College of Physical Education, Shenzhen University, Shenzhen, China; ^2^ College of Physical Education, Southwest University, Chongqing, China

**Keywords:** executive function, cognition, exercise, dual task, functional near-infrared spectroscopic

## Abstract

**Objective:** This study investigated the relationship between executive function and prefrontal cortex oxygenation during exercise in young adults with different Cardiorespiratory fitness (CRF) levels.

**Methods:** A total of 28 amateur runners (*n* = 14) and sedentary college students (*n* = 14) were recruited. The maximum oxygen uptake estimated for the sub-maximal intensity run (4.97 miles/h) was used to indicate the different CRF levels. After 1 week, participants must complete the Stroop and 2-Back tasks in silence while performing moderate-intensity exercise. Using 19-channel functional near-infrared spectroscopic (fNIRS) to examine changes in prefrontal cortex oxyhemoglobin.

**Results:** There was no significant difference in the correctness of the Stroop and 2-Back tasks between the two groups during exercise, but the amateur runner group showed an acceleration in reaction time. fNIRS results showed that during the exercise 2-Back task, the left dorsolateral prefrontal cortex oxyhemoglobin was higher in the amateur runner group than in the sedentary group.

**Conclusion:** Executive function during exercise was similarly improved in participants with better fitness, suggesting that CRF provides an excellent metabolic reserve and directed allocation for cognitive tasks during exercise.

## 1 Introduction

Running is the world’s most widely participated sport. In 2021, approximately 49 million people in the United States will have run or jogged at least once (Statista, 2023). Running reduces all-cause mortality risk and improves mental health, such as cognitive function (Hillman et al., 2008; Lee et al., 2014) and even leads to beneficial plastic changes in the brain (Cao et al., 2021). In addition, running is a complex physical activity that involves multitasking in many scenarios. For example, endurance athletes’ coping with fatigue during the competition (Behrens et al., 2023), and implementation of and decision-making about speeding strategies all require significant cognitive function involvement (Hyland-Monks et al., 2018). Simultaneously maintaining or sustaining motor and cognitive tasks is important in human life. It is necessary for daily, physical, vocational, and educational activities (Behrens et al., 2023).

Cognitive functions are the processes associated with specific brain areas (Pessoa, 2008). As a “higher” cognitive function, the executive function (EF) controls and regulates “lower” cognitive processes and goal-directed, future-oriented behavior (Alvarez and Emory, 2006). The brain’s prefrontal cortex (PFC) activity is the main driver of EF (Alvarez and Emory, 2006). Compared with other cognitive domains, EF appears to be more susceptible to the positive effects of various forms of movement (Spirduso, 1975; Schroeter et al., 2002; Tomporowski, 2003; Woo and Sharps, 2003; Hillman et al., 2006). For example, after acute exercise, EF and cortical activity performance in relevant brain areas can be improved (Byun et al., 2014). And during exercise, the response time to perform functions at low and moderate intensities becomes faster compared to the calm state (Davranche et al., 2015; Giles et al., 2018; Cantelon and Giles, 2021).

However, EF is susceptible to cardiorespiratory fitness (CRF). CRF reflects the integrated ability to transport oxygen from the atmosphere to the mitochondria to perform physical work (Ross et al., 2016). Studies have found that exercise-induced improvements in CRF are associated with better EF (Scott et al., 2016; Goenarjo et al., 2021). However, we know little about the performance of EF in dual-task running exercise states in different CRF participants during running exercise. Giles et al. (2018) found that endurance athletes improved response times to Stroop tasks during moderate-intensity exercise and observed a reduction in PFC and task-related oxy-Hb. Most participants currently focused on exercise—EF dual-task studies are physically active young adults, and there is a lack of comparisons between people at different levels of health (Cantelon and Giles, 2021).

Therefore, this study’s main aim was to explore EF’s performance during running exercises in adults with different CRFs. This study also uses functional near-infrared spectroscopic imaging (fNIRS) to observe cortex activity concerning EF tasks. fNIRS is a promising neuroimaging tool relatively insensitive to motion artifacts (Scholkmann et al., 2014; Ludyga et al., 2019; Yan et al., 2020). Portable fNIRS can be used in many complex scenarios and is widely used in sports science (Tempest and Reiss, 2019; Wu et al., 2022). This study hypothesizes that 1) only participants with high CRF will improve EF response time during exercise compared to a single task, and 2) participants with high CRF will have higher PFC activity during the running-EF task.

## 2 Materials and methods

### 2.1 Participants

We planned to recruit different groups to represent different CRF levels. The sample size calculation was first performed. The sample size was estimated using G-power 3.1 based on a previous study by Goenarjo et al. (2020b). The results showed that a minimum of twenty participants were needed. We expanded the sample size. Twenty-eight amateur runners and sedentary regular college students were recruited (amateur runners: *n* = 14, sedentary group: *n* = 14). The amateur runners were college athletes with an average of over 4 years of training. The International Physical Activity Questionnaire--Short Form (IPAQ-SF) screened the sedentary group. Each sedentary member was sedentary for no less than 8 h per day. Other basic physical indicators are shown in Table 1. In addition, all subjects met the following inclusion criteria: 1) right-handed; 2) no sports injuries within 6 months; 3) normal vision and no color blindness; 4) no neurological disorders such as head trauma or brain injury; 5) not using the antidepressant/anxiety medication; and 6) not required to exercise according to a doctor’s prescription. The Ethics Committee of Shenzhen University reviewed and approved this experiment (PN-202300017), and all the participants signed an informed consent form before participation.

**TABLE 1:** Basic information for participants at different fitness levels (Mean ± Standard deviation).

	Amateur runners’ group	Sedentary group	*p*
N	14 (8males, 6females)	14 (8males, 6females)	
Age (year)	22.43 ± 1.65	21.93 ± 1.59	0.422
Height (cm)	171.77 ± 7.76	168.82 ± 8.73	0.353
Wight (kg)	67.41 ± 12.86	60.21 ± 8.91	0.097
Physical Activity (MET-min/week)	4105.43 ± 4327.54	1053.30 ± 442.91	**0.003**
Sedentary time (hour/day)	2.46 ± 0.91	10.4 ± 2.38	**<0.001**
PSQI	10.00 ± 1.52	9.14 ± 1.88	0.502
DASS-Depression	2.57 ± 1.99	1.79 ± 2.72	0.587
DASS-Anxiety	2.50 ± 2.41	1.93 ± 2.09	0.181
DASS-Pressure	4.71 ± 3.43	3.00 ± 2.88	0.249
GSES	30.1 ± 0.57	26.7 ± 0.42	0.209
VO_2max_ (mL/kg/min)	Female: 48.50 ± 0.74	Female: 39.26 ± 2.42	**0.020**
	Male: 54.31 ± 1.16	Male: 44.80 ± 1.73	**<0.001**

PSQI, pittsburgh sleepiness scale; MET, the metabolic equivalent; DASS, depression, Anxiety and Stress Scale; GSES, General Self-Efficacy Scale; VO2max, Maximum oxygen uptake; Bolded indicates a significant difference between the two groups.

### 2.2 Study design

Participants were tested twice at different time points. They completed an informed consent form and a basic information questionnaire during the first session. The International Physical Activity Questionnaire Short Form was used to screen participants for sedentary time (Craig et al., 2003). The Depression, Anxiety, and Stress Scale (DASS) (Brown et al., 1997), the Pittsburgh Sleepiness Scale (PSQI) (Buysse et al., 1989), and the General Self-Efficacy Scale (GSES) (Cheung and Sun, 1999) were used to evaluate their psychological wellbeing. Participants then completed a cardiorespiratory fitness test by jogging on a treadmill at a given intensity (4.97 miles/h). In the second session, participants wore the fNIRS and performed the Stroop and 2-Back tasks in a quiet and moderate-intensity exercise state for behavioral and fNIRS data collection. The two sessions occurred >7 days apart to avoid the effects of fatigue. During this time, participants were instructed to maintain their daily routine and avoid engaging in extreme physical activity. After 7 days, the experimenter-initiated contact with subjects to participate in the second session. Participants were told to abstain from alcohol or beverages containing caffeine for 24 h before each test.

### 2.3 Measurement of CRF

As this study may have included an unfit group, we used a more conservative sub-maximal intensity run of a given intensity to estimate maximal oxygen uptake (VO_2max_). The George prediction method is considered reliable for predicting VO_2max_ (George et al., 1993). The method has many advantages such as low cost, no need to reach exercise limits, and no need for trained personnel. The test is determined by applying a constant load of 8 km/h (4.97 miles/h) on a treadmill. A heart rate belt (Polar Team System, Kempele, Finland) is used to check the heart rate. During the test, the runner starts at a self-selected pace, gradually accelerates to a set speed, and stops the test when a steady-state heart rate (SSHR) has been reached for no more than 3 min. The exercise heart rate is considered a steady state when consecutive heart rates (30-s interval) differ by ≤ 3 beats/min. VO_2max_ is estimated using the following formula:

$${VO}_{2\max}=54.07+7.062\times gender\;\left(0=female;1=male\right)-0.1938\times weight\left(kg\right)+4.47*speed\left(miles*h^{-1}\right)-0.1453*SSHR\left(bpm\right)$$

### 2.4 Cognitive tasks

The Stroop task and the 2-Back task were used to evaluate the execution function.

#### 2.4.1 Stroop task

**FIGURE 1:**
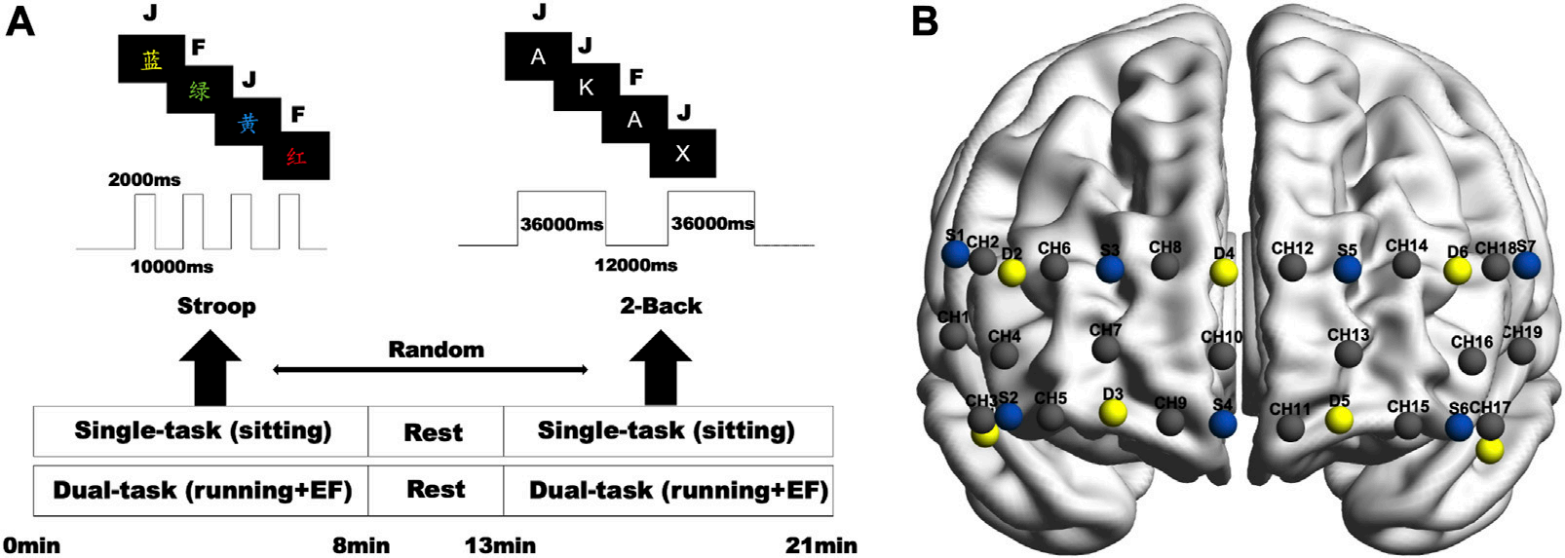
**(A)** Experimental procedure and task protocols. **(B)** fNIRS probe settings (blue = source; yellow = detector; gray = active channel).

For this experiment, we chose a computer-based color-word matching Stroop test with an event-related design implemented using E-Prime 3.0 software (Psychology Software Tools Inc., United States). Many neuroimaging techniques, including fNIRS, have used this classic measure to evaluate cognitive function in the prefrontal cortex (MacLeod, 1991). In the task, subjects observed the names of colors presented in different ink colors. In the inconsistent condition, the color names matched other ink colors (e.g., red words written in green ink). In the neutral condition, the ink color of the color name matched the meaning of the word itself (e.g., a red word written in red ink). A computer screen with a black background randomly shows letters of different colors. The participant must press the keyboard’s corresponding button as fast as possible (F for neutral, J for inconsistent). All words for this task were in Chinese, which consisted of 20 incongruent and 20 congruent experiments. Each word had a presentation time of 0.5 s, and the participant had to choose within 2 s, or it was considered an error. Subjects had a 10-s break between words (Figure 1).

#### 2.4.2 2-Back task

To evaluate working memory, we used a block-based 2-Back task. In each task block, an English capitalized consonant letter appeared in the middle on a black screen for 2 s with an inter-trial interval of 0.5 s. Participants are required to respond when the letter is the same as the previous letters. The participant presses F for unanimous and J for inconsistent. The output records response times and correctness rates. There are five blocks throughout 2-Back, each consisting of 12 trials lasting 36 s, with a 12-s interval between blocks. The percentage of correct responses and the response time are recorded (Figure 1).

### 2.5 Cognitive tasks during quiet and exercise

Behavioral experiments are conducted in a dark environment without interference from other moving objects. In the second session, participants randomly selected the Stroop task and the 2-Back task in two different states. Each cognitive task lasted approximately 8 min. Participants needed at least 30 s in the exercise state to reach a given heart rate before performing the 1-min cognitive task. There is a 5-min break in between. The dual-task behavior experiment takes place on a treadmill. During exercise, each subject wears a Polar heart rate detector to limit the intensity of the exercise. The experimenter controlled the treadmill’s speed according to the subject’s heart rate. The monitor was set approximately 1.2 m in front of the subject’s body and held in place with a special bracket. Participants held two extended numeric keypads (corresponding to F and J, respectively) that were long enough for the participants to perform cognitive tasks during the exercise. Before the official start, participants were allowed sufficient time to debug the display and keyboard until they reached the most comfortable position. Initially, participants walked at a self-selected pace and gradually transitioned to jogging. Participants began performing the task when the heart rate reached and stabilized at a moderate intensity (Garber et al., 2011). For the definition of exercise intensity, we adopted the recommendations of the American College of Sports Medicine’s Guidance for Prescribing Exercise. We used 64%–76% percent of maximum heart rate (%HRmax) as moderate exercise intensity (Garber et al., 2011). Maximum heart rate was calculated using the Gellish et al. optimization estimation equation: maximum heart rate = 207–0.7 * age (Gellish et al., 2007). The experimenter adjusted the treadmill’s speed according to the participant’s heart rate range to maintain it at a moderate intensity range.

### 2.6 fNIRS data acquisition and processing

In the experiments, the fNIRS device uses a multi-channel portable Nirsmart (Danyang Huichuang Medical Equipment Co., Ltd., China) to record changes in the signal of oxyhemoglobin (oxy-Hb). The device uses 7 light sources and 7 detectors with a distance of 3 cm between the light sources and the detectors and contains 19 active channels. Effective coverage of the frontopolar, dorsolateral, and ventral lateral prefrontal areas. Each light source emits light at 760 and 850 nm wavelengths, and all channels are set at 11 Hz. The locations of channels were located using a three-dimensional digitizer. The coordinates of the 19 channels on the Montreal Neurological Institute (MNI) standard brain were imported into the NIRS-SPM toolbox to obtain the distribution probability for each channel (Ye et al., 2009). Projection of MNI coordinates of light sources, probes, and channels determined by a 3D localizer onto the cortical surface of the ICBM152 brain template using MATLAB-based BrainNet (Xia et al., 2013) (Figure 1).

fNIRS data were analyzed using the Homer3 NIRS processing package. First, the raw optical data is converted to optical density data (Huppert et al., 2009). Channels with poor signal quality recorded at the beginning of each session during calibration and determined using the enPruneChannels function were excluded (dRange = 1e-03 1e+04, SNRthresh = 2, SDrange = 0–30). Secondly, the hmrR_ MotionartifactByChannel function (tMotion = 0.5, tMask = 1.0, STDEVthresh = 20, AMPthresh = 5.0) helps identify and exclude headache signals on a channel-by-channel basis to lessen the effects of motion artifacts. Third, perform wavelet-based motion correction (Molavi and Dumont, 2012). The data were converted to oxy-Hb concentrations using a modified Beer-Lambert equation after applying bandpass filters (standard cutoff frequencies of 0.001 and 0.2 Hz). The motion artifacts were then corrected using the MotionCorrectCbs function. After pre-processing, the general linear model calculates a beta estimate indicating the hemodynamic response during the task.

### 2.7 Statistical analysis

The data were statistically analyzed using SPSS 26.0. Using independent samples t-tests to compare physical and psychological variables between the two groups of participants. ANOVA with two-way (state × group) repeated measures was used to test the null hypothesis. Examine whether there are any main or interaction effects of groups (amateur runners or sedentary group) and state (single task or double task) on task performance. Bonferroni was used to correct the results of *post hoc* multiple comparisons in behavior. After excluding channels for pretreatment in the fNIRS results, a linear mixed-effects model (LMM) was used to compare differences between factors. Using the false discovery rate (FDR) to correct for multiple comparisons. All data are expressed as mean differences (MD), and effect sizes are reported as partial eta squared (*η*2) in the ANOVA.

## 3 Result

### 3.1 Participant characteristics

Table 1 summarizes the characteristics of the participants. *t*-test results showed no differences in mental health between the two groups (*p* > 0.05). Clearly, the amateur runners’ group had significantly greater MET than the sedentary group and less sedentary time than the sedentary group (*p* < 0.01). In addition, VO2max was higher in amateur runners of different genders compared to the sedentary group. These performances can distinguish between two different groups of CRF.

### 3.2 Behavioral results

#### 3.2.1 Stroop

**FIGURE 2:**
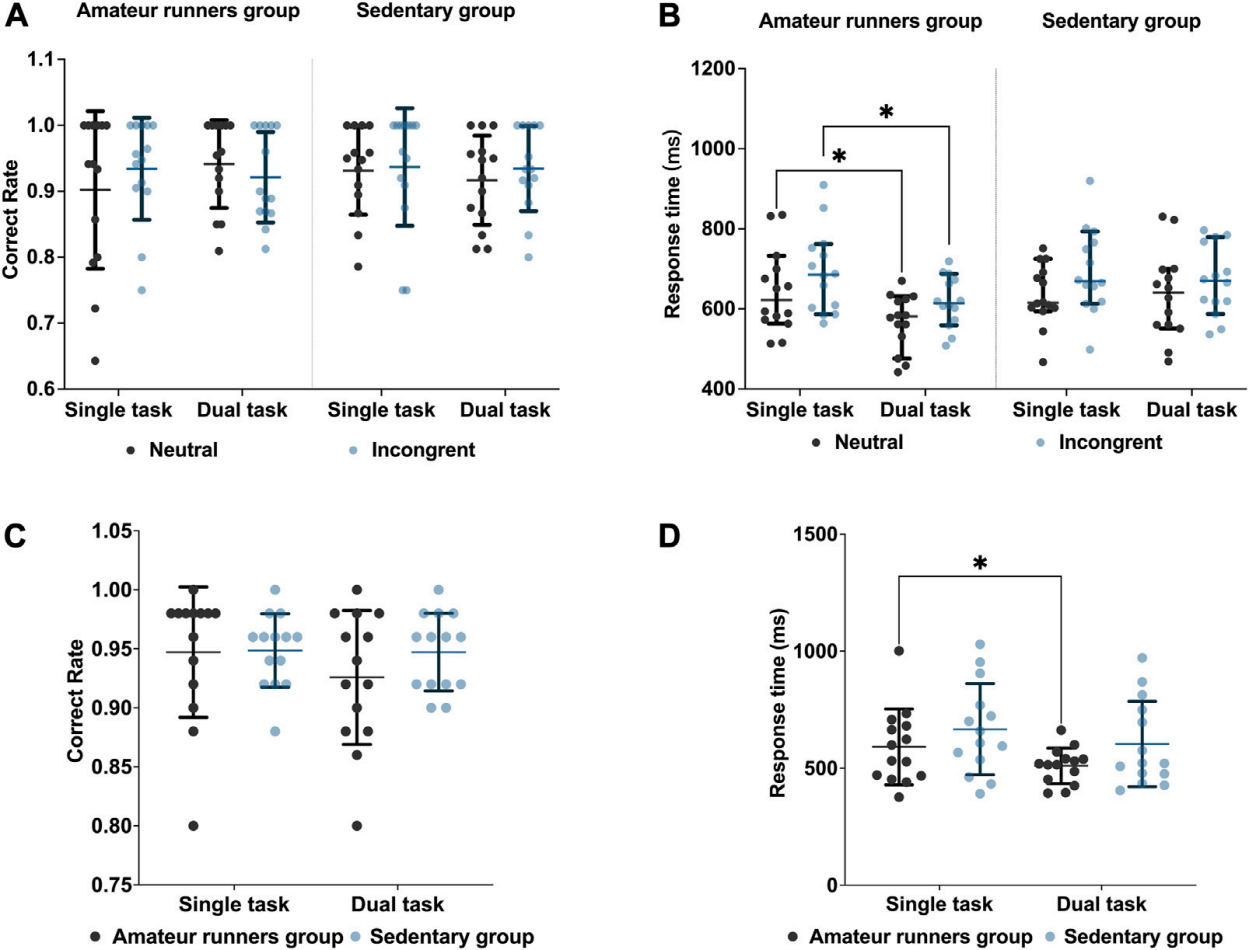
Results compared between the two groups: Stroop task: **(A)** correct rate; **(B)** response time. 2-Back task: **(C)** correct rate; **(D)** response time. Data are expressed as mean ± standard error. * Indicates a significant difference compared to the resting state. *p* < 0.05. Bonferroni corrected.

All behavioral and cognitive outcomes are presented in Figure 2. For correctness, there were neither inter-subject effects nor interactions [*F* (1, 26) = 1.530, *p* = 0.227, *η*2 = 0.056] among the group [*F* (1, 26) = 0.077, *p* = 0.784, *η*2 = 0.003], task condition [*F* (1, 26) = 0.268, *p* = 0.609, η2 = 0.010], and state [*F* (1, 26) = 0.049, *p* = 0.826, *η*2 = 0.002]. For reaction time, the main effects of consistency [*F*= (1, 26) = 31.333, *p* < 0.001, *η*2 = 0.547] and state [*F* (1, 26) = 10.051, *p* = 0.004, *η*2 = 0.279] showed significance. No interaction was found [*F* (1, 26) = 0.337, *p* = 0.567, *η*2 = 0.013]. Posthoc analyses showed that the amateur runners’ group improved in both neutral (*MD* = −75.35 *m*, *p* = 0.033, *Bonferroni* corrected) and inconsistent (*MD* = −74.06 *m*, *p* = 0.036, *Bonferroni* corrected) responses (Figure 2).

#### 3.2.2 2-Back

For correctness in the 2-Back working memory task, the results of the repeated measures ANOVA showed neither a main effect nor an interaction between group [*F* (1,26) = 0.589, *p* = 0.450, *η*2 = 0.022] and state [*F* (1, 26) = 1.724, *p* = 0.201, *η*2 = 0.062]. In comparison, for the response, the state played a significant main effect [*F* (1, 26) = 6.378, *p* = 0.018, *η*2 = 0.197]. Post-hoc analysis showed that only the amateur runners’ group had faster reaction times during dual-task (*MD* = −93.12 *m*, *p* = 0.043, *Bonferroni* corrected) (Figure 2).

### 3.3 fNIRS result

#### 3.3.1 Stroop

**FIGURE 3:**
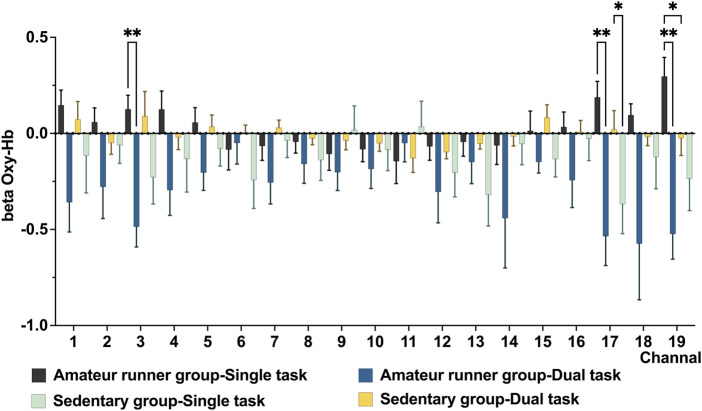
Compares prefrontal oxygenation in the Stroop task between the high-fit and low-fit groups in the quiet and exercise states. Data are presented as mean ± standard error. *: *p* < 0.05, **: *p* < 0.01, *FDR* corrected.

For the Stroop task, the main effect of state in the LMM results showed higher oxy-Hb in the amateur runner group in the single task state in CH3 (*MD* = −0.617, *p* = 0.006, *FDR* corrected), 17 (*MD* = −0.723, *p* = 0.008, *FDR* corrected). The sedentary group had significantly lower oxy-Hb levels in CH19 (*MD* = −0.820, *p* = 0.002, *FDR* corrected) and CH17(*MD* = −0.390, *p* = 0.030, *FDR* corrected) compared to the single-task state. Among the main effects of the groups, the amateur runners’ group had significantly higher oxy-Hb in CH19 in the single task state compared with the sedentary group (*MD* = 0.321, *p* = 0.025, *FDR* corrected). However, we did not find significant differences between the two groups in the dual task state. (Figure 3).

#### 3.3.2 2-Back

**FIGURE 4:**
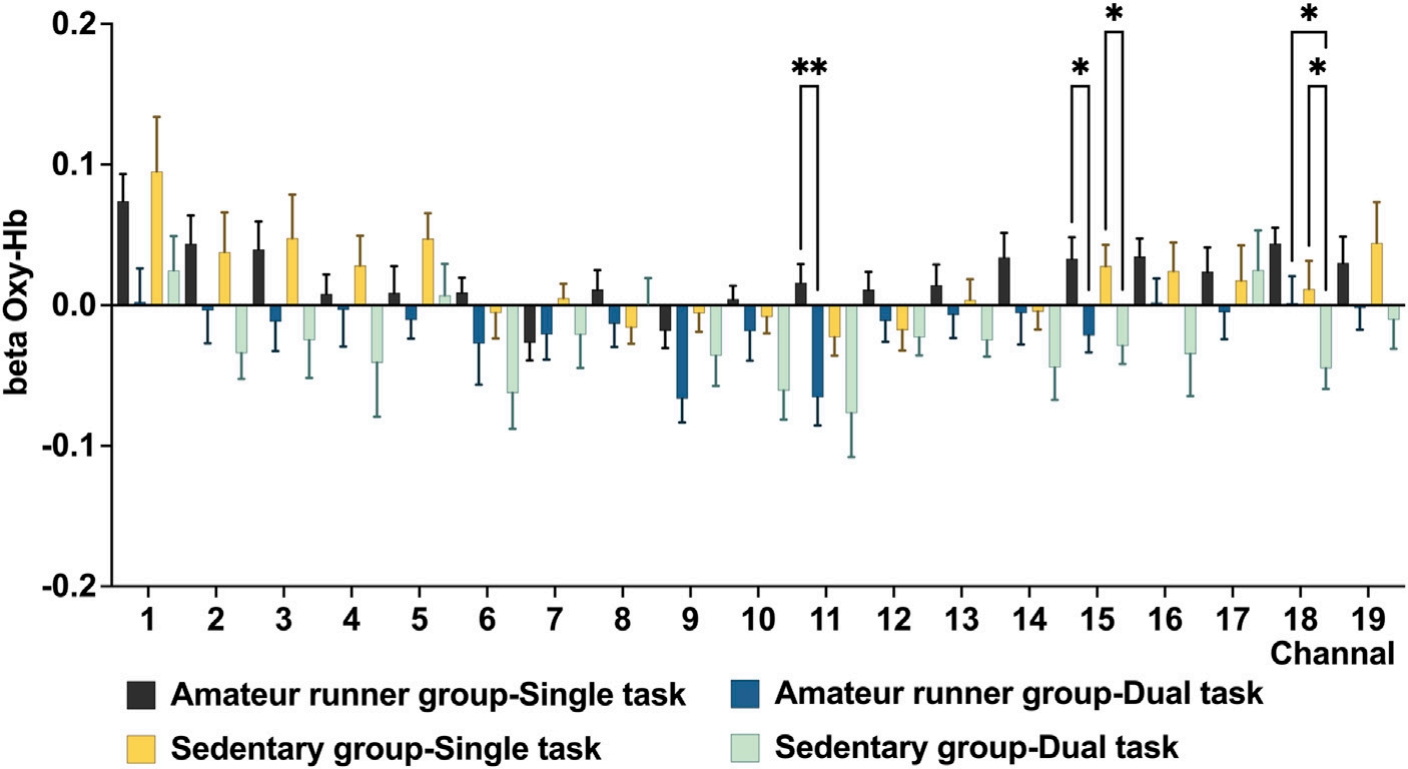
In the 2-Back task weight, changes in beta values of prefrontal 0xy-Hb in the amateur runner group and sedentary group in the single task and double task states. Data are presented as mean ± standard error. *: *p* < 0.05, **: *p* < 0.01, *FDR* corrected.

**FIGURE 5:**
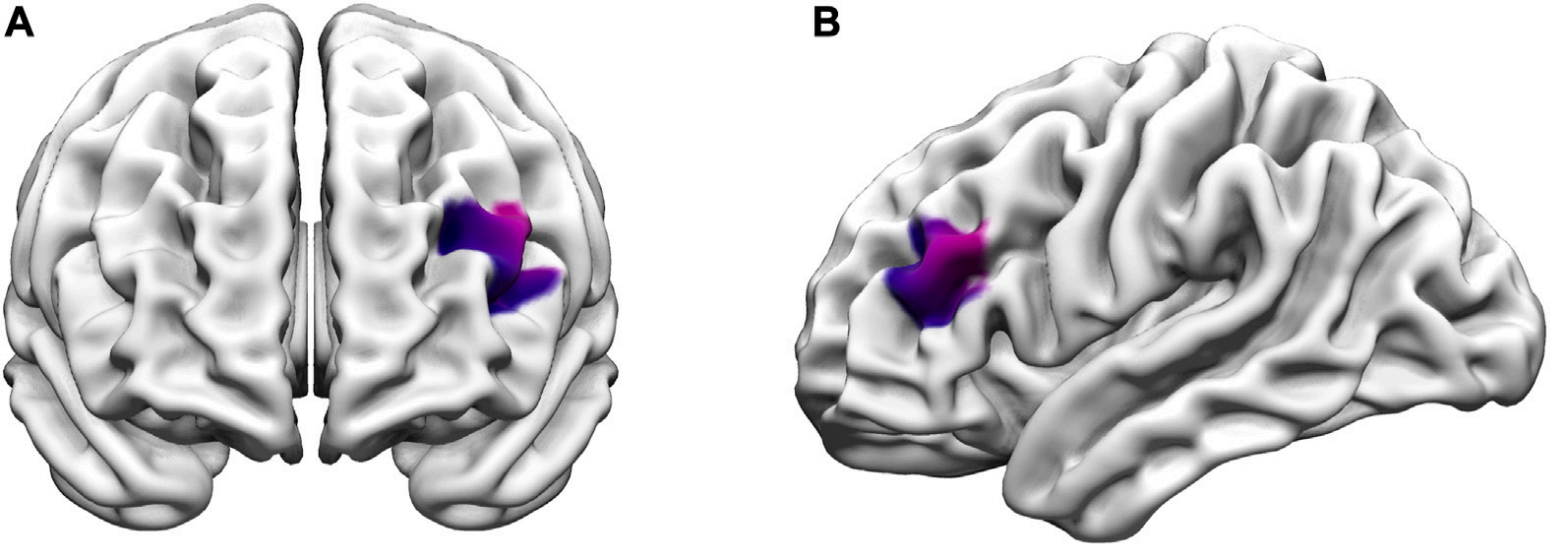
Differences in prefrontal oxy-Hb between the amateur runner and sedentary groups during the exercise-2-Back task. The purple color indicates the channels with significant differences (CH18). **(A)** Anterior view; **(B)** left view (*p* < 0.05, *FDR* corrected).

For the 2-back task, the main effect of state in the LMM showed that the amateur runners’ group had significantly lower oxy-Hb during dual-task in CH11 (*MD* = −0.08, *p* = 0.005, *FDR* corrected), and compared with the single task state, CH15 (*MD* = −0.05, *p* = 0.018, FDR corrected) had significantly lower oxygenated hemoglobin levels. In addition, compared with the single task state, the oxy-Hb levels in the sedentary group during exercise were significantly lower in CH15 (*MD* = −0.06, *p* = 0.018, *FDR* corrected) and CH18 (*MD* = −0.06, *p* = 0.018, *FDR* corrected) (Figure 4). In the group main effect, during exercise, the amateur runners’ group had significantly higher oxy-Hb in CH18 than the sedentary group (*MD* = −0.05, *p* = 0.018, *FDR* corrected). The channel was mainly in the left dorsolateral prefrontal lobe (Figure 5).

## 4 Discussion

This study aimed to explore EF performance and brain oxygenation during exercise in adults with different CRF. The results partially supported our hypotheses. Specifically, this study found amateur runners’ group participants showed improved reaction times in the Stroop and the 2-Back task. Still, there were no changes in accuracy in either group during the exercise. In addition, in the single EF task, participants with the amateur runners’ group had higher oxy-Hb in the left PFC during the single cognitive task. In the dual exercise-EF task, participants in both groups showed lower oxy-Hb in the PFC, but the amateur runners’ group had higher oxy-Hb in the DLPFC during the 2-Back task.

The above findings are consistent with previous meta-analyses, which found a slight improvement in performance on cognitive tasks during acute exercise (Lambourne and Tomporowski, 2010; Chang et al., 2012). Chang et al. concluded that when assessing cognitive performance during exercise, a slight positive effect occurred in physically fit participants but not in participants with a lower health level (Chang et al., 2012). Similarly, we observed an improvement in the high CRF group. Several recent studies found that endurance athletes and physically active participants had improved reaction times (Giles et al., 2018; Zheng et al., 2022). However, our results differed from those of other studies for the low-fitness group. Labelle et al. found that low-fitness individuals showed greater instability in an inhibitory control task (Labelle et al., 2013). Untrained individuals’ simple response patterns during light and vigorous exercise became more sluggish (Brisswalter et al., 1997). And began to lose attention at 60% of maximal heart rate intensity (Hüttermann and Memmert, 2014). In contrast, the attentional performance of exercise experts increases linearly with exercise intensity (Hüttermann and Memmert, 2014). Differently, our findings did not reveal impaired cognitive performance in the sedentary group. The factors responsible for the differences in results may be multifactorial, which may be related to the different CRF measurements, exercise duration, and intensity.

In addition, EF is more sensitive to changes in CRF (Hall et al., 2001). However, Goenarjo et al. (2020b) found no differences in task performance and prefrontal oxygenation during a dual walking-cognitive task. Cognitive performance can improve with exercise, but beyond a certain work intensity, exercise can lead to central nervous system deterioration (Tomporowski, 2003). Specifically, 64%–76% maximum heart rate intensity is more intense for low CRF individuals but favorable for high-fitness individuals because of their higher anaerobic thresholds (Wasserman et al., 1973; Strangman et al., 2002; Şekir et al., 2002). Our results are useful for guiding physical activity. It is thought that increasing Cardiorespiratory levels could improve cognitive performance by increasing prefrontal white and grey matter (Erickson et al., 2014). In conclusion, the relationship between cognitive tasks during exercise and fitness levels is becoming more apparent.

For fNIRS, many studies have observed a decrease in oxy-Hb of the PFC during re-motor cognition (Giles et al., 2018; Huang et al., 2019; Zheng et al., 2022). Zheng et al. (2022) found that during the dual task of motor cognition, there is a redistribution of cerebral blood flow between metabolic and cognitive resources. It is now well established that oxy-Hb content in the prefrontal lobe increases at low to moderate exercise intensities and stabilizes at vigorous exercise intensities (Maidan et al., 2021). We speculate that this may be due to the baseline hemodynamic response in different states. The oxy-Hb response to stimuli under motor and cognitive tasks had higher baseline values than the single task, which may have led to differences in the fitted hemodynamic responses.

The observed between-group differences suggest that more energy-efficient participants during exercise maintained relatively higher oxy-Hb and did not exhibit higher efficiency. This can be attributed to the complex neural regulation required when running on a treadmill, which requires significant metabolic resources and additional cognitive demands of the task (Maidan et al., 2021). Some studies suggest that elite athletes have higher cortical activity during tasks of high complexity (Hung et al., 2004; Endo et al., 2006). Similarly, young people who consume less oxygen under a given load will have a greater oxygen supply to the brain to replenish cognitive resources. Cognitive resource theory suggests that the abilities required for attention and information processing are a limited but renewable set of resources. And the ability to process multiple tasks simultaneously is limited. Exercise can promote angiogenesis and synaptogenesis, which in turn may improve cognitive performance (Swain et al., 2003). In contrast, low CRF participants may mobilize more resources to maintain exercise stability, and they allocate fewer cognitive resources to exercise (Chang et al., 2012). Running at moderate intensities differed more from daily physical activity for participants with low CRF, and this greater than self-determined given intensity was more likely to observe a decrease in oxy-Hb. Results from a study of brain oxygenation in outstanding Kenyan athletes showed a greater decline in PFC oxy-Hb in poorer performers (Santos-Concejero et al., 2017). Whereas a 5% change in intensity is sufficient to alter this stable change in cerebral oxygenation (Santos-Concejero et al., 2017). However, these findings were only observed in the 2-Back task, but not in the Stroop task. This may be because the block-based design of the task can exhibit a more pronounced hemodynamic profile.

This study has many limitations, which may account for the differences between our results and most previous studies. First, previous studies have found that individuals with higher VO2 peak and VO_2max_ and more physically active participants may show better executive function in a single task (Goenarjo et al., 2020a; Goenarjo et al., 2020b; Goenarjo et al., 2021; Salzman et al., 2022). However, no such differences were found when evaluated using the estimated VO_2max_. Therefore, the reliability of George’s prediction method in evaluating participants’ cognition compared to direct measurement of VO_2max_ still needs further exploration and replication. Second, there may be significant differences by gender and type of exercise in affecting cognition. Based on the different exercise styles, running exercises may require more cognitive resources because they involve postural control and balance (Cantelon and Giles, 2021). A previous meta-analysis showed a worsening of the mean effect of cognitive measures in the running-cognition dual task (Lambourne and Tomporowski, 2010). Third, it is possible that CRF is also associated with better executive functioning in women (Dupuy et al., 2015; Scott et al., 2016). But research on gender differences is still lacking. However, research on gender differences is still lacking. Finally, this study should be as cautious as possible in interpreting the results due to the small sample size. Future research may require larger sample sizes and replicated studies, further exploring the effects of CRF on cognitive performance during exercise in various ways, including gender and intervention.

## 5 Conclusion

Overall, this study strengthens the evidence for improved EF reaction times in moderate-intensity exercise. Furthermore, participants with better CRF had higher PFC activity during single and dual motor cognitive tasks. Enhancing CRF may reduce the cognitive resources required for the exercise and allocate more cognitive resources to concurrent tasks, such as EF. Our results may provide a theoretical reference for future research and advocacy for fitness activities in non-healthy populations.

## Data Availability

The raw data supporting the conclusion of this article will be made available by the authors, without undue reservation.
